# Early childhood education teachers’ search for balance between children’s developmental needs and the expectations of the curriculum

**DOI:** 10.3389/fpsyg.2026.1841421

**Published:** 2026-06-04

**Authors:** Emine Kaya

**Affiliations:** Faculty of Education, Adıyaman University, Adıyaman, Türkiye

**Keywords:** academicization, child development, curriculum balance, early childhood education curriculum, program pressure, teacher perceptions, teacher stress

## Abstract

**Introduction:**

Despite the global emphasis on and spread of early childhood education, it has shown a tendency to become more academic and less focused on develop mental needs. This study aims to understand the process of establishing balance among early childhood education teachers caught between children’s develop mental needs and the expectations of the curriculum.

**Methods:**

The research was designed as qualitative and used a case study design. The participant group consisted of 15 early childhood education teachers working in Adıyaman province, located in the Southeastern Anatolia Region of Türkiye. Research data were collected through a personal information form and a semi-structured interview form.

**Results:**

The results show that teachers value children’s developmental needs and try to make time for them. The findings reveal that teachers struggle to meet children’s developmental needs due to the intensive program, crowded classrooms, lack of materials, and demands from school administrators and parents.

**Discussion:**

Teachers agree that there is insufficient time for play and free activities for children within the intensive academic program flow. The pressure to complete the curriculum has negative psychological effects on both children and teachers. The research highlights the significant impact of program pressure in early childhood education on the psychological well-being of both children and teachers.

## 1 Introduction

Early childhood education has short- and long-term benefits in individual, social, and economic terms. These benefits include academic and cognitive development, social and emotional competencies, personality and self-esteem development, economic gains, healthy lifestyle behavior acquisition, and supporting the participation of parents in employment (Baek et al., 2025; Cannon et al., 2018; García et al., 2017; Giren and Öztürk, 2020; Lassassi, 2021). According to the Council of Economic Advisers (2014) report, every dollar invested in early childhood education yields a return of $8.60 in the long term. In this regard, it is seen that countries are increasingly prioritizing preschool education. According to the data, while the coverage rate in preschool education was 33% in 2000, this rate rose to 61% in 2022. The school enrollment rate in early childhood education reaches 85% in developed countries, but unfortunately drops to 20% in poor countries (UNICEF, 2022). The United Nations has emphasized that state parties are obliged to provide all children with the necessary early childhood education by 2030 (United Nations, 2015).

Despite its growing importance and prevalence in the global context, early childhood education is showing a tendency toward academicization, moving away from developmental needs in terms of quality. In this study, the concept of developmental needs refers not only to cognitive development but also to social and emotional competence, the process of self-directed learning, and in particular, a holistic approach that includes play-based learning (free play) and time allocated to child-initiated activities (Lunga et al., 2022; Rahmawati, 2025). However, today, preschool education institutions are considered a stage of preparation for primary school, and some countries implement intensive curricula focused on academic success while deprioritizing the developmental needs of children. For example, in South Korea, 47.6% of students under the age of 6 and 25% of students under the age of 2 take 5–6 h of private lessons per week in subjects such as mathematics, English, and science at Hagwons (Financial Times, 2025). In South Korea, children are left to cope with academic trauma at an early age through exams at ages 4 and 7 (Kim, 2025). In China, with the mindset of “don’t lose the race at the starting line,” children begin taking private lessons in mathematics, English, and the local language from the age of 3. In China, homework-related sleep deprivation begins as early as age 3 (O'Meara, 2016). Parents, driven by Fomo (fear of missing out) and the dynamism of the country’s education system, push their children into academic lockdown at an early age (Kim, 2025). Pushing these children, who are just beginning to walk and run, into this education frenzy negatively affects their physical, emotional, and cognitive development, causing them to face problems such as stress, anxiety, and burnout at an early age. Academicization is the tendency to regard early childhood education institutions as a preparatory stage for the next level of schooling and focus on intensive cognitive and structured programs instead of child-centered learning (Alcock and Haggerty, 2013). Bassok et al. (2016) describe kindergartens as new first grades, drawing attention to the changing nature of kindergartens and stating that this transformation in the nature of early childhood education has reached alarming proportions. Quality early childhood education must address children’s developmental and learning needs in a holistic manner. Quality is a process in which social and emotional competence is prioritized, and free play and child-initiated activities are valued (Kamışlı et al., 2020). At the same time, a delicate balance must be established between this approach and more structured, cognitively focused activities. Öngören (2023), Markova (2016), and Rupin et al. (2023) emphasized the supportive role of play-based pedagogy in social and emotional development and learning. Learning through play has been shown to increase motivation, create lasting learning experiences, and encourage active participation in the learning process. This demonstrates the importance of play-based learning in early childhood education.

Studies show that children raised in an intensely academic environment before school age do not have an academic advantage and that their creativity and emotional development are negatively affected (Hirsh-Pasek et al., 1990). In their study, Schweinhart et al. (1986) found that children who participated in early childhood education programs with academic and teacher-centered curricula had higher rates of behavioral problems, drug use, and vandalism during adolescence. Similarly, a longitudinal study revealed that academic pressure and expectations at an early age negatively affect the psychological well-being of classmates in later years (Shin, 2024). There are studies showing that exposure to intensive early childhood education leads to academic delay (Wang, 2024) and behavioral problems (Bi et al., 2024). Contrary to these studies, some studies argue that academic gains in early childhood education are not lost but may actually increase (Ansari, 2018; Bai et al., 2020; Horm et al., 2022). Melhuish (2011) found that individuals who received early childhood education at an early age had higher levels of education, income, and socioeconomic status at age 28 compared to other students.

The trend toward academicization in early childhood education affects not only students but also teachers. In the process of early academicization, students are exposed to intensive academic activities, desk-based work, and portfolio assignments instead of play, exploration, and free-play activities. Preschool children, who are pressured to perform tasks intended for older age groups, initially experience excitement but later feel anxiety. Under this pressure, children miss out on important developmental milestones (Elkind, 2001). Early childhood education teachers, while aware of children’s pedagogical needs, find themselves caught between the expectations of national curricula and the pressures from parents and school administrators. Academically focused education places pedagogical demands on teachers and leads to variations in practice among them. Intensive academic education programs weaken child-centered education in classrooms. In this process where academic success takes precedence over children’s developmental needs, teachers’ experiences and perspectives are considered significant.

This study, which aims to reveal how early childhood education teachers balance children’s developmental needs with curriculum expectations and the challenges they face in trying to achieve this balance, addresses the following sub-questions.

1. How do teachers establish a balance between the social, emotional, and play-based needs of children and cognitive learning goals in the context of intensive curricular expectations?
2. What are the main problems experienced by teachers in meeting the play and free time needs of children while implementing the curriculum and the strategies they develop to cope with these problems?
3. What is the perceived degree of flexibility and autonomy of teachers in their control over the content, ordering, and duration of implementing the curriculum?
4. What are the sources of curriculum pressure perceived by teachers? How does this pressure affect the implementation strategies adopted to maintain the development of children?

This study focused on the early childhood education policies and practices in the province of Adıyaman in the Southeastern Anatolia Region of Türkiye, which may involve organizational and curricular differences, and experiences of teachers in this context. It is believed that the research will guide the development of more child-centered and applicable programs by revealing the balance teachers try to establish between children’s developmental needs and program expectations. Therefore, it is expected that the research results will shed light on school administrators in terms of supporting teachers’ pedagogical decisions and shifting towards student-centered practices, as well as on teachers who want to overcome difficulties in their professional practices. The study is expected to guide parents by revealing the effects of the trend toward academicization at an early age on students from the teachers’ perspective. It is anticipated that it will guide policymakers in developing more applicable policies regarding maintaining the balance between program expectations and children’s developmental needs in early childhood education. This study, which addresses the effects of academicization tendencies in early childhood on teachers and teacher practices, is expected to contribute to the field of early childhood education. It is also anticipated that it will provide data to international institutions for shaping early childhood education programs in developing countries and contribute to the grounding of education programs within the framework of children’s rights for civil society organizations and education associations. Furthermore, it is expected to make a unique contribution to studies on early academicization in an international context and, given the international nature of the topic, to offer a comparative perspective with other countries. The original data obtained from the Southeastern Anatolia Region of Türkiye will contribute to the national literature while also providing comparative information for international contexts facing similar issues. By revealing the balancing strategies of teachers, this study offers unique, evidence based, and guiding contributions to both theory and practice.

## 2 Method

### 2.1 Research design

This study was designed using the single-case study design, one of the qualitative research methods. The case study focuses on the classroom reflections of the early academicization process within the context of preschool education in Adıyaman Province and the balancing practices experienced by teachers during this process. The scope of the case study is strictly limited to official educational institutions in the central city and villages of Adıyaman Province in terms of location, and to the 2025–2026 academic year in terms of time. This study is a regional case study aimed not at producing generalizable findings but at deeply understanding teachers’ experiences within a specific context. The study examines the effects of the early academicization process on teachers, the dilemmas experienced in daily practice, and the strategies teachers have developed on their own, based directly on teacher interviews. A case study, was deemed appropriate for this study as it aims to understand participants’ experiences in detail and develop a multidimensional perspective (Cohen et al., 2021; Yin, 2003).

### 2.2 Participants

The participant group of the study consisted of 15 early childhood education teachers working in early childhood institutions affiliated with Adıyaman province during the 2025–2026 academic year. Considering that Adıyaman is located in the Southeastern Anatolia Region of Türkiye, and the socio-cultural structure of this region may create potential differences in curriculum implementation, this province was chosen to examine in depth the regional dynamics of the search of teachers for balance. Participants were selected through maximum diversity sampling. Maximum diversity sampling was used to select individuals who were different in terms of the research topic, with the aim of adding a broad and in-depth perspective to the study (Cohen et al., 2021; Patton, 2001). The diversity here focuses not so much on geographical distribution as on variations in participants’ years of professional experience (2–18 years), class sizes (7–27 students), and the types of institutions where they work (village schools, district centers, and provincial centers). This approach has made it possible to conduct an in-depth examination of the internal heterogeneity and regional dynamics of the Adıyaman case. This strategy deliberately included individuals with different demographic and professional characteristics (e.g., age, professional seniority, class size) that could affect the examined topic (early academicization pressure and the search for balance), ensuring that the phenomenon was understood from a broad and in-depth perspective. Table 1 shows the demographic characteristics of the participating teachers.

**Table 1:** Demographic characteristics of the participant group.

	Gender	Age	Years of experience	Place of work	Class size
P1	Female	45	16	Province/Center	10
P2	Women	41	18	Province/Center	20
P3	Women	36	13	Province/Center	19
P4	Women	40	14	Province/Center	27
P5	Women	35	13	Province/Center	27
P6	Women	34	4	Province/Center	26
P7	Women	37	2	Village	15
P8	Female	27	4	Village	19
P9	Women	28	3	Village	8
P10	Women	40	14	Province/Center	21
P11	Women	36	5	Province/Center	25
P12	Female	36	14	Village	7
P13	Female	37	13	Province/Center	25
P14	Female	35	14	Province/Center	19
P15	Women	33	4	District center	20
Average		37.33	9.93		19.2

### 2.3 Data collection tools

Research data was collected through a personal information form and a semi-structured interview form. The personal information form consisted of questions about the teachers’ age, gender, professional seniority, class size, and place of work. The semi-structured interview form was prepared by the researcher and consisted of seven questions.

When preparing the semi-structured interview form, the literature related to the research topic was first reviewed. Eight semi-structured questions were prepared for the form. The questions were sent to four academics specializing in educational programs and teaching, early school education, Turkish language teaching, and psychological counseling and guidance for review. In addition to experts in early childhood education and curricula, experts in the fields of Turkish language teaching and psychological counseling and guidance were consulted to ensure that the questions addressed not only content appropriateness but also linguistic accuracy and potential sensitivities regarding the psychological development of preschool children. An analysis of Question 8 in the draft questionnaire (Teachers’ approaches to managing academic expectations in parent communication processes) revealed that this question showed a high degree of semantic overlap with Question 4 of the questionnaire, which concerns curriculum pressure and coping strategies. To preserve the construct validity and focus of the data collection tool, this question was removed from the form, and the interviews were conducted based on the remaining 7 questions. A pilot study of the interview form, which was reduced to 7 questions, was conducted with 2 early childhood education teachers. As a result of the pilot study, the interview form was found to be ready for implementation after evaluating the comprehensibility, duration, and content appropriateness of the questions. The questions covered the core balance themes specified in the sub-questions of the study, such as the emphasis of teachers on activities supporting social and emotional development, their perceptions of the time allocated to play and free time, the flexibility of the program, the difficulties encountered in responding to individual needs, and strategies for coping with curricular pressure.

### 2.4 Data collection process

The interviews were conducted in an empty room at the teachers’ own schools to make them feel comfortable. To maintain the researcher’s impartiality and non-directive approach, all teachers were asked the questions in the same order, and the researcher focused solely on listening without judging or directing the responses of the participants. Additional leading questions were avoided, except in cases requiring clarification. The interview process lasted between 40 and 50 min for each teacher. The average duration was calculated as 44 min. To determine whether data saturation had been reached, an assessment was conducted concurrently with the analysis process. Starting with the 12th interview, it was observed that no new themes or codes emerged and the existing data began to repeat itself; however, the process was completed with the planned 15 participants in order to confirm the depth of the data. The interviews were recorded. The audio recordings were transcribed word for word, and the written text was confirmed by the teachers.

### 2.5 Data analysis

To examine the narrative-based responses of the participants in an orderly manner and reveal recurring patterns in the data, it was deemed necessary to analyze the qualitative data obtained in the study in a systematic manner. This is why the content analysis method was used to analyze the data. The analysis began with a comprehensive reading of the transcripts. The transcripts were read a second time and then coded. Themes and sub-themes were created as a result of the coding. Logical consistency was taken into account when creating themes, and the codes were reviewed repeatedly. After the coding process, the opinion of an academic expert in the field of measurement and evaluation in education was sought to ensure inter-coder consistency. This academic coded all transcripts independently of the researcher. The consistency between the researcher’s and the expert’s codes was calculated using the Miles and Huberman (1994) formula, and the inter-coder agreement rate was found to be 92%. The expert not only reviewed the final themes but also contributed to the transparency of the analysis process by independently coding the raw data (transcripts). The themes were: the importance given to social and emotional development and its implementation; factors affecting social and emotional development; perceptions of time allocated to play and free time; efforts and methods to protect play time; difficulties in responding to individual needs; time management methods for coping with difficulties; criteria for deciding to change activities; methods for changing activities; the level of flexibility of the program, the level of control of the teacher, encountering developmentally inappropriate demands, methods of protecting and coping with the interests of children, the perception of program pressure and its sources, the effects of parenting pressure on children, coping with pressure, and methods of managing.

To enhance the transparency of the analysis process, Table 2, which shows how the initial codes were transformed into themes, is presented in this section. Table 2 demonstrates how the initial codes identified through detailed readings were converted into the themes used in the findings section.

**Table 2:** Teachers’ social and emotional development practices.

Theme	Category	Code	Participants	f
Importance and application of social and emotional development	Level of importance	High importance/Prioritization	P1, P2, P3, P4, P5, P6, P7, P9, P10, P11, P13, P14, P15	13
	Frequency and duration of implementation	Daily/Frequent application	P1, P7, P11, P15	4
		Weekly/Periodic application	P5, P9, P10	3
		Specific time frame	P8	1
		Frequency based on progress status	P14	1
Factors affecting social and emotional development	Supportive factors	School management support	P1, P10, P12, P14	4
		Parent understanding/Participation	P1, P5, P10, P14	4
		Program flexibility	P4, P7, P12, P15	4
		Child readiness	P14	1
	Limiting factors	Class size (Overcrowded)	P2, P3, P4, P5, P6, P7, P10, P13, P14, P15	10
		Material shortage	P1, P7, P8, P9, P12, P13, P15	7
		Intense program pace	P5, P14, P15	3
		School administration requests/Additional tasks	P2 (specific days/weeks), P4 (various tasks), P9 (intensive project tasks)	3
		Parent expectations/Disinterest	P2 (academic expectations), P3 (parental attitudes), P8, P9, P14 (disinterest)	5
		Inadequacy of the classroom environment/Physical Conditions	P2, P4	2
		children’s different learning levels	P14	1
		Irregular class attendance	P12	1
	Effects of restrictive factors on implementation	Implementation challenges	P1, P2, P3, P4, P5, P7, P8, P10, P11, P12, P13, P14, P15	13
		Loss of motivation/Burnout	P2, P14	2

### 2.6 Ethical considerations

Ethical approval for the study was obtained from the Adıyaman University Social Sciences and Humanities Ethics Committee with the decision numbered 190 and dated 09/09/2025. The specific measures taken to protect the ethical rights of the participants were as follows: An informed consent form was obtained from all participants prior to the interviews. The identities of the participants were kept confidential. The participants were informed that they had the right to stop the interview or withdraw from the study at any time. The data were anonymized throughout the article using the codes P1, P2, … P15 instead of participant names. Interview recordings and transcripts were stored and managed securely (in an encrypted file) for academic purposes only.

## 3 Findings

### 3.1 Findings regarding teachers’ social and emotional development-focused practices

Within the scope of the first sub-problem, teachers were asked, “When preparing your activity plans, how much importance do you place on supporting children’s social and emotional development? How often and for how long do you include such activities? Also, what factors support and constrain you when prioritizing social and emotional development? What can you say about the effects of these factors on your daily practice?”. The findings regarding the views of the participants are presented in Table 2 under the data analysis heading to demonstrate how the data coding process was carried out.

When the codes are examined, it is seen that 13 of the teachers attach great importance to social and emotional development. Teacher P2 expressed the importance they attach to students’ social and emotional development and the factors that limit them in this regard with the following statements.

P2: “For me, children’s social and emotional development is very important. Early childhood education has a special significance in a child’s life. I consider it very important to support their social and emotional development during this period when they take their first steps in their educational life. Unfortunately, there are factors that limit me in this regard. These include large class sizes, the endless demands of the school administration, especially activities expected on certain days and weeks, the classroom environment not being prepared in a way that supports children’s social and emotional development, and parents’ academic expectations. No matter how much I try to ignore these, I feel overwhelmed at times.”

### 3.2 Findings regarding the extent to which children’s needs for play and free time are met in the program

Within the scope of the second sub-problem, the first question asked to teachers was: “Do you think there is enough time in the daily flow for children to play, have free time, and discover their own interests? Do you make a special effort to preserve playtime?” Teachers’ approaches to play and free time activities are given in Table 3.

**Table 3:** Teachers’ approaches to play and free time.

Theme	Sub-theme	Code	Teacher codes	f
Perception of time allocated for play and free time	Perception of sufficient time	Thinks there is enough time left	P1, P3, P6, P11, P12, P14	6
	Perception of insufficient time	Thinks there is not enough time left	P2, P4, P5, P7, P8, P9, P10, P13, P15	9
Efforts and methods to protect play time	Using program flexibility	Adjusting/Shortening activity durations	P1 (pay attention to time management), P2 (covering the topic briefly/superficially), P14 (time management, superficial application), P15	4
		Prioritizing/Allocating play time	P5 (30-min game duration, does not give up even if it does not finish)	1
		Evaluating remaining time	P7 (after feeding/short activity)	1
	Environment and content adjustments	Creating environment and materials	P3 (environment and materials according to areas of interest)	1
		Exploring interests through different activities	P4 (using techniques within activities, even if not during free time)	1
		Open-ended/Free play support	P8 (open-ended in art activities), P9 (extending free time, supporting children’s own play in the garden at), P10 (enriching unstructured/semi-structured play)	3
		Toy rotation	P13 (different toy groups each day)	

Emphasizing the insufficiency of playtime, P2 stated that they had to take time away from children’s playtime at home by giving them homework when necessary. P5 stated that even though they knew the program would not be completed, they never neglected playtime and allocated at least half an hour a day for play. P13 emphasized that they played with different toy groups every day so that children would not get bored while playing. P15, who believes playtime is insufficient, expressed their thoughts with the following statements.

P15: “I think the time is insufficient. In the daily routine, I make a special effort to ensure that children play and have free time. Because play is the most natural way for them to both learn and discover themselves. Even if the program is intense, I am careful not to cut playtime. If necessary, I shorten or extend activities. The interest children show during play gives me the most realistic clues about their development.”

Teachers were asked the following question as the second sub-question under the second sub-problem: “What difficulties do you experience in recognizing and responding to children’s individual needs amid a busy schedule, time pressure, or tasks that need to be completed? What methods help you in terms of time management?” Teachers’ views on the difficulties they experience in responding to individual needs and their practices in time management are presented in Table 4.

**Table 4:** Challenges and solutions in individualized education.

Theme	Sub-theme	Code	Teacher Codes	f
Difficulties in responding to individual needs	Program and time-related difficulties	Intense program flow/Time pressure	P1, P2, P8, P9, P14, P15	6
		Extra tasks	P8, P9	2
		Rigidity of class durations	P8	1
	Classroom and student-related difficulties	Large class size	P2, P3, P6, P7, P10, P15	6
		Student management and coordination difficulties	P2	1
		Student behavior problems	P3	1
		Material shortage	P13	1
		Lack of support staff	P7	1
	Difficulties arising from parental expectations	Parents’ academic expectations	P2	1
Methods for coping with difficulties and time management	Planning and preparation	Progressing according to plan	P1, P3, P5, P10	4
		Making preparations	P4, P5	2
	Individual focus and flexibility	Observing children	P10, P11, P12	3
		Minimizing the program/Prioritizing social–emotional development	P13	1
		Organizing the activity flow	P14, P15	2
		Advantage of student numbers	P12	1
		Giving the child the opportunity to cope on their own	P7	1
	Overtime	Overtime/Taking work home	P4, P14	2

Teachers stated that they experienced time constraints due to the pressure of a busy schedule and extra tasks assigned to them (on specific days and weeks, preparing bulletin boards, etc.). P8 stated that there are no breaks in early childhood education in Türkiye and that they are forced to teach for 300 min without interruption. Complaining about this situation, they emphasized that their motivation as teachers had decreased. P7 stated that they work in a village, have 15 students in their class, and have to do everything themselves because there is no support staff in the classroom, which takes up their time. P4, who stated that they work overtime to avoid time management issues at school, expressed themselves with the following sentences.

P4: “My priority is to ensure that children communicate effectively and feel safe. I try to minimize stressful situations by staying at school longer after class and doing extra preparation at home.”

The third question posed to teachers under the second sub-problem was: “When you have to cancel some activities during implementation or change them according to the children’s immediate needs, how do you make this decision? What criteria do you use to decide on these changes?” Teacher decision criteria and methods for activity changes are given in Table 5.

**Table 5:** Teacher decision criteria and methods for activity modification.

Theme	Subtheme	Code	Teacher Codes	f
Activity change decision criteria	Child’s developmental status and needs	Readiness	P1, P4, P13, P15	4
		Interest, motivation, and curiosity	P2, P3, P9, P12, P15	5
		Level appropriateness	P6	1
		Development level	P5	1
		Learning process	P12	1
	Program and external factors	Program flexibility	P4, P10	2
		Material/Environment conditions	P2, P3, P7	3
		Special circumstances (Teacher/Child)	P1	1
		Specific days and weeks	P2	1
		Staying within the scope of learning outcomes	P14	1
Activity modification methods	Content/Duration adjustment	Lightening the topic/Alternative activity	P5, P6, P15	3
		Integrated activities	P8	1
		Repetition and deepening	P2, P9	2
		Offering options	P4	1
	Postpone/Cancel	Canceling/Postponing the event	P7	1
		Spreading topics	P8	1
	Student participation	Joint decision making	P3, P10	2
		Children determining the process	O11	1

Teachers emphasized that when making changes to activities, they often took into account the children’s readiness, emotional state, interests, desires, and needs. Teachers underscored that they replaced activities they did not find appropriate for their students’ levels with alternative activities, repeated them from time to time, and postponed the topic if necessary. Teachers stated that they delved deeper into and focused on the parts of the topic that aroused curiosity in students. They also emphasized that weather conditions, lack of materials, and the classroom environment played a role in changing activities. Some teachers lightened the subject or applied more interesting activities without going beyond the daily learning outcomes.

### 3.3 Findings regarding teachers’ perceptions of flexibility and freedom in decision-making during program implementation

The first question posed to teachers under this heading was, “In your opinion, does the current early childhood teaching program allow enough flexibility for implementers, i.e., teachers? How much control do you feel you have over the content, sequence, or duration of activities?” Teachers’ opinions about program flexibility and implementation controls are shown in Table 6.

**Table 6:** Program flexibility and implementation control.

Theme	Sub-theme	Code	Teacher Codes	f
Program flexibility level	Finding flexibility insufficient	Program rigidity/Intensity	P1, P2, P5, P7, P8, P9, P11, P12, P13, P15	10
	Finding the flexibility adequate	Thinking the program is flexible	P3, P4, P6, P10, P14	5
Teacher’s level of control	Difficulty/Limitations in control	Difficulty in controlling duration and time	P1	1
		Difficulty in controlling a crowded classroom	P5, P13	2
	Control	Making changes to content/Order/Duration	P2, P3, P8, P14, P15	5
		Providing flexibility based on your own situation	P12	1

When examining teachers’ responses, the view that the program is intensive and rigid is widespread. The fact that all details in the program, such as subject matter, duration, and activities, are meticulously calculated by the ministry bothers some teachers. Teachers emphasize that there is no flexibility in the program and that they are not working under humane conditions due to the effort to complete the 300-min uninterrupted program. Some teachers, however, stated that they only found the program inflexible in terms of time, but otherwise partially flexible. P15 emphasized that no matter how restrictive the program was, it was implemented without deviating from the main points. Direct teacher comments regarding program flexibility are provided below.

P1: “I think the current early childhood education program does not allow teachers flexibility. Sometimes I find it difficult to control the duration of activities and time management.”

P9: “I don’t think it provides enough flexibility. I think it’s too focused on teaching. There should be more play and freedom.”

Under this heading, the second question posed to the teachers was: “Do you sometimes encounter demands that you do not find developmentally appropriate for children, based on management, parents, or institutional policies? How do you deal with such situations and how do you protect the children’s interests?” Teachers’ attitudes and practices towards inappropriate requests are given in Table 7.

**Table 7:** Teachers’ attitudes and practices in response to inappropriate requests.

Theme	Sub-theme	Code	Teacher codes	*f*
Encountering developmentally inappropriate demands	Source of demands	Requests originating from management/Institutional policies	P2, P7, P8, P9, P15	5
		Parent-originating demands	P2, P6, P7, P8, P12, P14, P15	7
Methods for protecting children’s interests and coping	Refusing/Withdrawing requests	Rejecting with clear communication	P1 (emphasizing that it will not be fulfilled, setting boundaries), P11 (direct refusal)	2
		Refusing by providing a reason	P4 (explaining the reason for non-compliance), P13 (withdrawing the request using appropriate language), P14 (explaining why it is not appropriate)	3
		Emphasizing children’s priority	P5, P15 (indicating that children are the priority)	2
		Do not apply until there is a chance of rejection	P10 (refuse if there is a possibility of refusal, otherwise meet the minimum requirement)	1
		Do not apply if not appropriate	P2	1
	Communication and guidance	Parent communication and cooperation	P3, P8, P12, P14	4
		Principle of equality	P6	1
	Request Conversion/Simplification	Simplification according to the child’s level	P9	1

Teachers stated that ministry policies and school administration policies made their jobs difficult. They stated that they constantly had to prepare programs for specific days and weeks, such as Mother’s Day and Local Products Week, that comparisons were made between classes at school, and that if they did not do so, they were portrayed as bad teachers. In a way, they emphasized that they were forced to carry out these practices. They also added that parents overwhelm them with their instinct to put their own child first (P6). P15, who always emphasized that their priority was the children, expressed their thoughts with the following sentences.

P15: “Yes, sometimes I encounter requests that I don’t find developmentally appropriate for the children. In such cases, I first try to explain my own observations and the children’s needs to the administration and, if necessary, to the parents. I explain with examples why it is more appropriate to take a different approach, focusing on the child’s best interests. It usually works, if not always. When necessary, I make adjustments to the program, taking care to both meet expectations and protect the children’s development. Most importantly, I always make my decisions based on the principle of ‘the child’s best interests.’”

### 3.4 Findings on teachers’ perceptions of program pressure and implementation strategies

Finally, teachers were asked, “Does the intensity of the early childhood education curriculum sometimes force you to rush to complete the program? Have you observed any negative effects of this situation on children’s social, emotional, or cognitive development? When you encounter pressure to complete the program, what methods do you use to reduce this pressure and its possible negative effects?”. Teachers’ perception of program pressure and coping strategies are shown in Table 8.

**Table 8:** Teachers’ perception of program pressure and coping strategies.

Theme	Category	Code	Teacher Codes	f
Perception of program pressure and its sources	Pressure felt due to program intensity	Feeling pressure	P15, P1, P12, P13, P2, P4, P7, P8, P9	9
		Balancing/Postponing pressure	P11, P14, P5, P4	4
		Does not feel pressure	P10, P3, P6	3
	Additional limiting factors that increase pressure	Class size/Environment	P15, P1, P12, P7, P5	5
		Additional tasks/Paperwork	P2, P7, P8	3
The effects of parenting pressure on child development	Negative effects on cognitive and learning processes	Superficial/Quick transition	P1, P13, P14, P9, P15, P7, P8	7
	Negative effects in the social, emotional, and motivational domains	Boredom	P15, P12, P2, P8, P13	5
		Restriction	P15, P2, P8	3
		Sense of inadequacy	P2, P14	2
	No negative effects observed	Not observed	P10, P3, P6	3
Coping with pressure and management methods	Revision in focus and time management	Child-centeredness	P15, P11, P14, P5, P9	5
		Flexibility/Procrastination	P15, P13, P4, P5	4
		Planned progress	P4, P5, P3	3
	Converting activity content	Simplification/Abbreviation	P15, P2, P5	3
		Gamification/Breaks	P15, P13, P2, P12	4
		Review/Simplification	P14, P9	2
	Revision and external support request	Program Revision request	P15, P8	2
		Parent guidance	P13	1

When examining the table, it is seen that the majority of teachers emphasized that the program forced them to rush, and that rushing caused children to try to move on to the next stage without learning (P14). P13 emphasized that rushing causes children to neglect their social, emotional, and cognitive development. In contrast, P3 stated that with good planning, they could master the program, while P10 stated that the program did not force them to rush. Teachers stated that not only the program load but also additional tasks and paperwork exhausted them. Teachers stated that when they taught subjects under pressure to cover the material, they covered it superficially and quickly (P7, P8, P15), were unable to do enough reinforcement activities, and observed attention deficits, motivation problems, and boredom in children. P13 mentioned that preschool children are playful children and that such an intensive program negatively affects them. P12 stated that they constantly do desk activities in crowded classrooms, even if they do not want to, and that this makes them sad.

P2: “Of course I think about it. The pressure to complete the program stresses me out a lot. Sometimes I can’t finish the activity I do in class. I give the rest of the activity to the children as homework. Unfortunately, I can’t do this when we need to spend a lot of time on the activity. This negatively affects the children. The children are very young. Their lives are actually built around play. We are doing them an injustice by sitting them down at a desk all day, giving them crayons, or doing activities that are above their cognitive level.”

Teachers state that the most common and effective method for coping with program pressure is to apply the principle of child-centeredness (P5, P9, P11, P14, P15). P15 states that when he feels pressure, he creates a positive classroom atmosphere by prioritizing the needs of the student group. P5 states that they do not care about the pressure to achieve in the program and enjoy teaching. P2 states that they try to cover some activities more superficially and in a shorter time due to time constraints, while P13 states that when they see the negative effects of rushing on children, they give them short play breaks. P14 emphasizes that they cover topics gradually in a simple way and try to repeat them often. Teachers requested a program revision to address these issues.

## 4 Discussion and conclusion

This study examined how early childhood education teachers strive to balance program expectations with children’s developmental needs. Findings indicate that teachers prioritize students’ social and emotional development. Teachers emphasize their awareness that early childhood is a unique period and that they prioritize children’s developmental needs during this time. Teachers were seen to implement practices aimed at meeting children’s developmental needs on a daily, weekly, or monthly basis. This is consistent with the literature on quality early childhood education supporting a holistic approach and is considered important in that it reveals the effort teachers make to preserve this holistic approach. Although teachers emphasized children’s social and emotional development, they noted that there were certain factors that constrained them. Among these factors, teachers most frequently mentioned large class sizes. They emphasized that large class sizes make it difficult to respond to individual needs, cause difficulties in implementation, and lead to loss of motivation. Teachers working in large classes reported experiencing more difficulties, while teachers working in smaller classes stated that they were able to make better use of program flexibility. It was observed that the pedagogical practices of teachers working in low-enrollment classes in villages (T12, class size 7) differed from those of teachers working in crowded classes in city centers (T5, class size 27). Furthermore, a lack of materials and an unsuitable physical environment constrain teachers. The findings show that teachers’ pedagogical focus is disrupted due to external demands and additional tasks. Although teachers care about students’ developmental needs, they are forced to deviate from ideal practices due to demographic and environmental reasons. A review of the literature reveals studies with similar findings to this one. Blatchford et al. (2003), Blewitt et al. (2021), and Lasi et al. (2023) concluded in their studies that group sizes and time constraints limit teachers’ social and emotional pedagogy. Konstantopoulos and Shen (2023) found a linear relationship between reduced class size and interpersonal skills. Similarly, Humphries et al. (2018) stated that time constraints, paperwork, and academic demands are barriers to children’s social and emotional development. Otwate et al. (2025) and Rupin et al. (2023) stated that one of the biggest barriers to learning through play in early childhood is the lack of play materials. Nordin and Mohamed (2023) revealed that the biggest obstacles teachers face in implementing playtime are time constraints, lack of materials, and insufficient support from administrators and parents. Although the studies were conducted in different countries around the world, the similarity of the results is noteworthy. The results of this study reveal the necessity of increasing the number of teachers and classrooms to bring classroom sizes in line with humane conditions. It is also clearly evident that preschool curricula should be simplified, and their heavy subject load should be reduced.

The search for balance at the heart of the research is supported by teachers’ views (*f* = 10) that it stems from the intensive and rigid nature of the education program. Teachers emphasized that they felt compelled to rush due to concerns about adapting to the program. In addition to program pressure, the physical environment was observed to create additional pressure on teachers. Teachers added that the pressure to complete the program limited play and free time activities the most. Teachers stated that they shortened the duration of activities in the program or included play and free time activities in the remaining mini time slots in order to make time for play and free time. Numerous studies in the literature highlight the importance of play for child development. Öngören (2022) emphasized that play supports social and emotional development in early childhood. Similarly, Rupin et al. (2023) highlighted the contribution of play to learning and its supportive effect on social–emotional development through a more flexible program and activities tailored to children’s interests. Markova (2016), who emphasizes the positive effect of play on academic development, focused on the impact of non-academic free play on English language teaching in the early childhood education period. In this context, it is thought that increasing play and free activities in early childhood education programs will support teachers’ efforts to strike a balance. The results provide unique information for both theory and practice in the context of revealing the balancing strategies of teachers.

Research findings reveal that teachers clearly observe the negative effects of intense program pressure on children. Teachers listed the effects of pressure as cognitive negativity and social and emotional problems. Teachers are concerned that children will miss important developmental milestones in their lives. They emphasized that children experience boredom, overwhelm, and feelings of inadequacy due to intense program pressure. Anderson and Perone (2024) emphasized the importance of parents recognizing boredom in early childhood and teaching ways to cope with it. Studies have shown that children who spend a large part of their day at school squeezed between intense program pressure and academic tasks may be prone to negative behaviors such as substance abuse, reckless risk-taking, alcohol and internet addiction, etc. in later years (Biolcati et al., 2017). Jones et al. (2015) stated that emotional and social competence in children supports academic achievement. Academic development and social and emotional development influence each other and should be evaluated with a holistic approach. It is clear that the cognitive, social, and emotional effects of program pressure should not be overlooked. At this point, the content, flexibility, quality, and suitability of the program for the age group play a decisive role in protecting children from negative effects that may arise in the short and long term. In this context, it is thought that the results of this study will guide parents in understanding the negative effects of the early academicization process on children (e.g., boredom, feelings of inadequacy, socio-emotional problems).

The findings show that teachers act according to the philosophy of putting the child at the center as a coping strategy when they feel pressured. It is also seen that they try to protect children’s development by simplifying activity content or shortening it in some cases. It is clear that teachers manipulate the program flow in a way. Furthermore, it is seen that they adopt different approaches when faced with demands from school administrators or parents that are contrary to the developmental needs of children. In this regard, the teacher’s temperament also causes them to adopt different approaches. While some teachers directly reject inappropriate demands with clear statements, others choose to reject them by explaining their reasons. Whitlock et al. (2023) emphasized in their study that teachers were unable to give sufficient space to play-based practices due to curriculum and teaching demands. Roche et al. (2024) stated in their study that when teachers were pressured by different sources such as the program, school administration, and parents, they tried to reduce the pressure by simplifying activities and reducing playtime. Frydenberg et al. (2021) show that teachers working in early childhood education try to protect children’s needs regardless of the direction of the pressure. Meesak et al. (2025) emphasized the critical role of parental factors in shaping academic skills in early childhood. Zinsser et al. (2014) study also shows that teachers care about children’s social and emotional development and emphasize the importance of parental guidance in early childhood education. This study also found that teachers value parental guidance to support students’ family and emotional development.

Teachers’ program pressure, need for flexibility, and coping strategies can be said to stem from factors such as age, professional seniority, and class size. For example, Teacher 5, who is 35 years old, has 13 years of professional seniority, and teaches a class of 27 students, expressed sadness at not being able to involve all children in activities. In contrast, P12, who is 36 years old, has 14 years of professional seniority, and a class size of 7, emphasized that she believes the program allows sufficient time for the developmental needs of the children. P9, who has lower professional seniority (3 years of professional seniority), stated that despite being younger and less senior, she felt the structural intensity of the program. P15 stated that she had great difficulty coping with the pressure of the program due to her low professional seniority (4 years) and a class size of 20-. In addition to all this, it is noteworthy that P4, who is 40 years old, has 14 years of professional seniority, and a class size of 27, stated that she kept up with the program by working overtime, despite her high seniority, which highlights the intensity of the program.

## 5 Conclusion

Research findings indicate that teachers find the early childhood program too intensive and believe it does not fully meet the developmental needs of children. Teachers try to minimize the pressure of the program and the negative effects of external factors through their own efforts. Teachers, who are tasked with balancing parents, school administration, educational programs, and educational policies, try to meet children’s developmental needs by using their autonomy in the classroom. The results of the study offer a comparative perspective for other countries undergoing similar academicization processes and hold the potential to provide data to international institutions for shaping the curricula of developing countries.

### 5.1 Limitations of the research and recommendations

The research is a qualitative case study. The participant group of the research consists of 15 early childhood education teachers working in Adıyaman province. Teachers from different geographical regions were not included in the research. This makes it difficult to generalize the data to all early childhood education teachers in Türkiye. The data is limited to the questions in the semi-structured interview form and the answers given to these questions. The research results are based solely on the perceptions and statements of the teachers who participated in the study.

Based on the findings of this study, concrete recommendations for policy and practice are presented. At the level of National Education Policy, preschool programs should be simplified, and their heavy subject load should be reduced so that children’s developmental needs are prioritized. A national standard can be developed by increasing the number of teachers and classrooms to bring classroom sizes in line with humane conditions, while deficiencies in the physical infrastructure and materials of schools can be remedied through local and global support programs. Pedagogical and psychosocial support can be provided to teachers at the level of school administrators and practitioners, which would make it easier for teachers to cope with curriculum pressure and make decisions that safeguard the best interests of the child. Mechanisms can be established to maintain the balance between curriculum expectations and the implementation of the “best interests of the child” principle through guides and activities for school management, teachers, and parents. In curriculum development processes, the views of teachers implementing these curricula can be systematically obtained, and functioning feedback mechanisms that collect feedback from the field can be strengthened. In terms of research and international recommendations, the long-term effects of practices can be examined in longitudinal studies in the future, and more comprehensive studies can be conducted with parents and school administrators. At the international level, child-centered preschool practices can be encouraged. Future studies may focus on different dimensions of the early academicization process by drawing on the limitations of this study. In particular, it is considered necessary to examine the reasons for the gap between policy and practice in depth. Furthermore, by ensuring that the data collection process is not limited to the perceptions of teachers, data can also be collected from children using drawing or interview methods to obtain a more enriched perspective on the effects of curriculum pressure on their social, emotional, and cognitive development. Although each country’s sociology, economy, and education system are different, children’s developmental needs are the same everywhere. This fact must not be overlooked.

## Data Availability

The datasets presented in this article are not readily available because the data will not be shared with anyone. Requests to access the datasets should be directed to eminekaya@adiyaman.edu.tr.
